# School Performance and the Risk of Suicidal Thoughts in Young Adults: Population-Based Study

**DOI:** 10.1371/journal.pone.0109958

**Published:** 2014-10-27

**Authors:** Kyriaki Kosidou, Christina Dalman, Peeter Fredlund, Cecilia Magnusson

**Affiliations:** 1 Centre for Epidemiology and Community Medicine, Stockholm County Council & Public Health Epidemiology, Department of Public Health Sciences, Karolinska Institutet, Stockholm, Sweden; University of Vienna, Austria

## Abstract

Although low school performance is related to attempted and completed suicide, its relationship with suicidal thoughts has been less clear. We conducted a population-based study including 10081 individuals aged 18–29 years in Stockholm, Sweden, and found a clear positive gradient in the risk of lifetime suicidal thoughts with decreasing levels of compulsory school leaving grades. This relationship was somewhat attenuated but remained significant in multivariate models accounting for family background, severe adult psychopathology and adult socioeconomic conditions. School failure is associated with an increased risk of experiencing suicidal thoughts and may also increase the tendency of acting upon them.

## Introduction

Recent studies have found a strong positive association between low school performance and the risk of attempted and completed suicide later in life [1]–[3]. However, evidence on the relationship between school performance and suicidal thoughts, the immediate precursor of suicide plans and attempts, is equivocal. One study among New Zeeland young people found school success to protect from suicidal thoughts, but multivariable models suggested that this association was fully explained by family background, early psychopathology and history of sexual abuse [4]. Another study from Australia found an association between school failure and attempted suicide but not suicidal thoughts among adolescents [5]. However, a recent study found that fair or poor perceived academic performance independently increased the risk of suicidal thoughts and attempts in Korean adolescents [6]. A study of female twins found that those who spent fewer years in education had an increased risk of developing suicidal thoughts [7]. However, this association was attenuated in multivariate models controlling for childhood adversity, personality and psychopathology. Finally, studies on the association between IQ, which is highly correlated with school performance [8], and suicidal thoughts have yielded contradictory results [9]–[11]. Some studies have found a positive association between low scores in intelligence tests and the risk of experiencing suicidal thoughts later in life [9], [11]. By contrast, higher IQ was associated with an increased risk of suicidal thoughts in adolescent males in a recent study [10]. There are no large population studies on the association between school performance and suicidal thoughts in young adulthood. Thus we studied school performance in relation to suicidal thoughts in a large sample of young Swedish adults.

## Method

We conducted a population-based study including 10081 individuals aged 18–29 years, residents in Stockholm County that participated in the 2002 and 2006 survey waves of the Stockholm Public Health Cohort [12]. The Stockholm Public Health Cohort is a population-based cohort study that has been established within the framework of Stockholm County Council public health surveys to inform on determinants and consequences of significant contributors to the current burden of disease. These surveys are undertaken every four years for health and risk factor surveillance purposes, and the data collected are used in policy formulation, planning and resource allocation by policy makers and health care planners. The 2002 and 2006 surveys involved random samples of the adult population of Stockholm County and data was collected using postal (2002) or postal and web-based questionnaires. Self-reported data was linked to data from a range of Swedish national administrative and health data registers using the unique personal identification number assigned to each Swedish citizen at birth and to immigrants upon arrival in the country. Response rates were 54% in 2002, and 51% in 2006. Compared with Stockholm county census data, men, those under the age of 45, those born outside Sweden, those single or separated, those unemployed and those with a low income were overrepresented among the non-responders [12]. Study participants with missing data were excluded leaving 8039 individuals in the final analytical sample.

### Ethics Statement

Written informed consent was obtained from all the study participants, and the Stockholm regional ethical review board granted ethical approval for the study.

### Main exposure

School performance was defined as grade point averages in the final year of compulsory education (year 9, when participants were approximately 16 years old), retrieved from the National School Register (available at http://www.skolverket.se.), and categorized into quintiles according to year of graduation.

### Outcome

Lifetime suicidal thoughts were assessed by the question: ‘Have you ever been in the situation that you seriously considered taking your own life, maybe even planned how you would do that?’. There were four answer alternatives: ‘No, never’, ‘Yes, in the last week’, ‘Yes, in the last year’, and ‘Yes, earlier than a year ago’. The three latter composed our outcome of having had lifetime suicidal thoughts.

### Covariates

Parental socioeconomic status (SES) (unskilled workers, skilled workers, lower non-manual employees, intermediate non-manual employees, higher non-manual employees, self-employed, defined as highest of the mother or father) was ascertained via the Swedish Population and Housing Census in 1990 and completed by self-reports. Parental education (compulsory, upper secondary, higher education, highest of the mother or father) and immigration status (native Swede, European and Non-European first-generation immigrant, European and Non-European second-generation immigrant) was obtained from the Longitudinal Integration Database for Health Insurance and Labour Market Studies (LISA) at Statistics Sweden (http://www.scb.se). The National Patient Register, held by the National Board of Health and Welfare, was used for obtaining information on hospital admissions for mental disorders (any diagnosis in Chapter V of ICD-8 and -9 or Chapter F of ICD-10) in study participants and their parents. A positive psychiatric history was defined as any admission prior to completing the surveys in participants, and at any time in parents. Financial strain (no financial strain, sought financial help from others, sought social benefits) and employment status (student, employed, unemployed, other) were self-reported.

### Statistical analyses

Analyses were conducted using SAS version 9.1 (SAS Institute Inc., Cary, N.C., USA). In order to increase statistical power for the analyses, the 2002 and 2006 samples were pooled. There were 76 individuals that had participated in both surveys, and these were excluded from the analyses. The final analytical sample of 8039 participants comprised 4311 men and 5770 women. We used calibration weights to reweight for non-response [12], [13]. Weights were designed by Statistics Sweden to re-calculate the population structure of Stockholm County with compensation for systematic non-response, created on the basis of available auxiliary variables from national registries and their association with survey data. The auxiliary variables included sex, age, country of birth, civil status, income, educational level, sickness allowance and area of residence. We carried out conditional logistic regression analyses to estimate crude and adjusted odds ratios (OR) and their 95% confidence intervals (CI) of lifetime suicidal thoughts in relation to school performance. We fitted a series of progressive multivariable regression models to adjust for age and sex (Model 1), immigrant status, parental SES, parental education and parental history of inpatient psychiatric care (Model2), occupation and financial strain (Model 3), and individual history of inpatient psychiatric care (Model 4). The Log Likelihood Ratio Test was performed to test whether the association of school performance with suicidal thoughts differed with sex.

## Results

The prevalence of lifetime suicidal thoughts was 23.2% (18% among men and 27% among women). 5.6% of the study participants reported experiencing suicidal thoughts in the last year, 1.1% during the last week and 16.5% earlier than a year ago. The prevalence of lifetime suicidal thoughts was similar in the two samples (23% in 2002 and 23.3% in 2006, t-test p = 0.742)

School performance was associated with the risk of lifetime suicidal thoughts in a graded manner (Table 1). ORs ranged from 2.09 (95% CI 1.79–2.44) for those in the lowest grade quintile to 1.64 (95% CI 1.41–1.92), 1.38 (95% CI 1.18–1.61) and 1.09 (95% CI 0.92–1.28) for those in the 2nd, 3rd and 4th quintiles respectively, after adjustment for sex and age (Model 1). The relationship was somewhat attenuated when controlling for family background (Model 2) and further attenuated when employment status and financial strain were added in the model (Model 3). In the final model (Model 4), ORs increased stepwise with a more than 50% increase in risk for those in the lowest grade quintile as compared to those in the highest.

**Table 1:** Odds ratios (OR) and 95% confidence intervals (CI) of lifetime suicidal thoughts in relation to compulsory school leaving grades among young adults aged 18–29 in the Stockholm Public Health Cohort.

	No of cases/non cases	Model 1* OR (95%CI)	Model 2¶ OR (95%CI)	Model 3† OR (95%CI)	Model 4‡ OR (95%CI)
***Grade point average***					
Lowest quintile	465/1104	2.09 (1.79–2.44)	1.94 (1.64–2.28)	1.63 (1.37–1.93)	1.56 (1.31–1.85)
2^d^ quintile	426/1258	1.64 (1.41–1.92)	1.60 (1.36–1.88)	1.46 (1.24–1.72)	1.45 (1.23–1.71)
3^d^ quintile	368/1272	1.38 (1.18–1.61)	1.35 (1.15–1.58)	1.27 (1.08–1.49)	1.27 (1.08–1.49)
4^th^ quintile	314/1284	1.09 (0.92–1.28)	1.08 (0.92–1.27)	1.06 (0.89–1.24)	1.05 (0.89–1.24)
Highest quintile	288/1260	1	1	1	1

* Model 1: Adjusted for age and sex.

¶ Model 2: Model 1 further adjusted for parental socioeconomic status, parental education, immigrant status and parental history of inpatient psychiatric care.

† Model 3: Model 2 further adjusted for employment status and financial strain.

‡ Model 4: Model 3 further adjusted for individual history of inpatient psychiatric care.

The results of using recent suicidal thoughts (i.e. suicidal thoughts experienced in the last week or in the last year) compared to lifetime suicidal thoughts as the outcome were similar, with most of the magnitudes of effects being slightly lower and the CIs larger with recent suicidal thoughts. ORs for recent suicidal thoughts were 2.08 (95% CI 1.62–2.67) for those in the lowest grade quintile, 1.38 (95% CI 1.06–1.80), 1.28 (95% CI 1.18–1.61) and 1.01 (95% CI 0.77–1.34) for those in the 2nd, 3rd and 4th quintiles respectively (Model 1).

The association between school performance and suicidal thoughts did not differ between men and women (χ2 = 4.76, df = 3, p = 0.31 for the log-likelihood test on the school performance×sex interaction).

## Discussion

In this large population-based study, we found a clear positive gradient in the risk of lifetime suicidal thoughts with decreasing levels of compulsory school leaving grades among young adults. Our study raises some points for consideration.

Firstly, the relationship between school performance and suicidal thoughts was attenuated, but remained significant, when controlled for financial strain and employment status, two indicators of adult socioeconomic conditions. Thus, although school failure hampers the obtainment of good employment and material resources, which are important for mental health [14], these mechanisms appear to only partly explain the relationship between low school performance and suicidal behaviour.

Secondly, studies on the relationship between low school performance and the risk of attempted and completed suicide have generally indicated stronger relationships than those for suicidal thoughts found in our study [1], [3]. This was true also for participants in the Stockholm Public Health Cohort [2]. According to a recent study, school performance was strongly associated with the risk of attempted suicide among young adults in this cohort [2], with a more than two-fold increase in the risk of attempted suicide among participants in the lowest level of compulsory school leaving grades as compared to those in the highest. Thus, low school performance may not only increase the risk of experiencing suicidal thoughts but also the tendency of acting upon them.

Cognitive function may be an important explanatory factor of the link between school performance and suicidal thoughts, especially in light of the graded relationship noted in these data. However, findings of the association between IQ and suicidal thoughts are inconsistent [9]–[11]. A study from Australia reported an inverse association between higher scores in some, but not all, forms of intelligence tests in childhood and the risk of suicidal thoughts later in life [9]. By contrast, higher IQ was associated with an increased risk of experiencing suicidal thoughts in adolescent males in a recent study [10]. Lastly, a study on IQ and the risk of suicidal thoughts found no positive association with incidence but with persistence of such thoughts in adulthood [11]. The authors concluded that the association between low IQ and self-harm may be because people with low IQ experience suicidal thoughts for more prolonged periods than those with high IQ or because they are more likely to act upon them. However, limited sample sizes [9], [11] and the possibility of selection bias [10] may have hampered the results in previous studies and explain the inconsistencies.

Finally, although we adjusted for social background, attained socioeconomic position and parental as well as individual adult psychiatric history, other mechanisms that explain the low school performance and suicidal thoughts relationship, such as childhood abuse or psychopathology and substance abuse, cannot be ruled out.

The large population-based sample and availability of extensive self-reported as well as prospectively collected register-based data are two strengths of this study. Limitations include attrition, albeit accounted for to some extent by calibration, possible measurement error in self-reports of suicidal thoughts and lack of information on some potentially confounding factors. Furthermore, the possibility of inverse causality cannot be ruled out since we do not account for childhood suicidal behaviour that may have influenced school performance. However, the relationship persisted even when recent suicidal thoughts were used as the outcome. Thus, low school performance appears to be of importance for developing suicidal thoughts also in adult life.

In conclusion school failure appears to predict suicidal thoughts among young adults and this is not explained by early socioeconomic conditions, severe adult psychopathology or later job attainment. Low school performance may also increase the tendency of acting upon suicidal thoughts.
